# Therapy-Induced Modulation of the Tumor Microenvironment: New Opportunities for Cancer Therapies

**DOI:** 10.3389/fonc.2020.582884

**Published:** 2020-10-23

**Authors:** Sergi Benavente, Almudena Sánchez-García, Silvia Naches, Matilde Esther LLeonart, Juan Lorente

**Affiliations:** ^1^Radiation Oncology Department, Vall d'Hebron University Hospital, Barcelona, Spain; ^2^Biomedical Research in Cancer Stem Cells Group, Vall d'Hebron Research Institute (VHIR), Universitat Autònoma de Barcelona, Barcelona, Spain; ^3^Otorhinolaryngology Department, Vall d'Hebron University Hospital, Universitat Autònoma de Barcelona, Barcelona, Spain; ^4^Spanish Biomedical Research Network Centre in Oncology, CIBERONC, Barcelona, Spain

**Keywords:** radiotherapy, immunotherapy, tumor microenvironment, surgery, cancer

## Abstract

Advances in immunotherapy have achieved remarkable clinical outcomes in tumors with low curability, but their effects are limited, and increasing evidence has implicated tumoral and non-tumoral components of the tumor microenvironment as critical mediators of cancer progression. At the same time, the clinical successes achieved with minimally invasive and optically-guided surgery and image-guided and ablative radiation strategies have been successfully implemented in clinical care. More effective, localized and safer treatments have fueled strong research interest in radioimmunotherapy, which has shown the potential immunomodulatory effects of ionizing radiation. However, increasingly more observations suggest that immunosuppressive changes, metabolic remodeling, and angiogenic responses in the local tumor microenvironment play a central role in tumor recurrence. In this review, we address challenges to identify responders vs. non-responders to the immune checkpoint blockade, discuss recent developments in combinations of immunotherapy and radiotherapy for clinical evaluation, and consider the clinical impact of immunosuppressive changes in the tumor microenvironment in the context of surgery and radiation. Since the therapy-induced modulation of the tumor microenvironment presents a multiplicity of forms, we propose that overcoming microenvironment related resistance can become clinically relevant and represents a novel strategy to optimize treatment immunogenicity and improve patient outcome.

## Introduction

Cancer treatment modalities vary considerably depending on stage and location, however surgical excision and radiation therapy are an integral part of treatment for most solid tumors. In an era of exceptionally dynamic evolution of knowledge, some recently published clinical studies have reshaped the role of surgery such as neoadjuvant immunotherapy combinations leading to less invasive surgery for advanced melanoma, antiangiogenics as an alternative to immediate surgery in renal cell carcinoma or upfront treatments making surgery possible for more patients with pancreatic cancer (1). Most therapeutic combinations in clinical trials are based on knowledge of resistance mechanisms and recently immunotherapy, which has revolutionized the clinical management of multiple tumors, has been included in multiple clinical trials which are mainly based on T cell and pursue a maintained antitumor immune response. Accumulating evidence suggests that conditioning the tumor microenvironment (TME) toward an immunomodulatory state may have a major impact on cancer outcome (2, 3). However, the TME comprises all the non-malignant cellular and non-cellular components of the tumor, including the immune system, blood cells, endothelial cells, fat cells, and the stroma. The tumor stroma is a critical component of the TME with cancer-promoting capacity as part of the response to treatments and leads to cancer resistance. For example, immunosuppressive cytokine secretion and metabolic alterations strongly participate in the suppression of host immune responses against tumor cells facilitating tumor proliferation. Extensive work exploring the interactions between cancer cells and the TME has been done but the advancements still require a better understanding of the potential targets before implementation in conceptual antitumor strategies. In this regard, recent advances resulting in more effective and localized radiation treatments (stereotactic radiosurgery and stereotactic body radiotherapy, SRS/SBRT) can achieve an effective alteration and ablation of tumor stromal tissue, which can be a singular advantage against tumoral immune evasion [reviewed in (4)]. In addition, technological developments have led to minimally invasive surgery with evident clinical benefits in terms of less invasiveness, excellent outcomes, and a shorter hospital stay (5).

In this review, we address challenges to identify responders vs. non-responders to the immune checkpoint blockade (ICB), discuss recent developments in combinations of immunotherapy and radiotherapy for clinical evaluation, and consider the clinical impact of immunosuppressive changes in the TME in the context of surgery and radiation. Overcoming microenvironment related resistance may have a fundamental impact on treatment efficacy and patient outcome.

## Characterizing the Immune Function in the Response to Checkpoint Inhibitor Immunotherapy

### Combinatorial Approaches to Treat Differences in the Immune Contexture of the TME

Immunotherapeutic approaches have transformed treatment and outcomes for some solid tumors, in particular, melanoma and non-small cell lung cancer (NSCLC), but do not benefit the majority of patients with cancer and have failed to induce broadly durable responses. Immunotherapy with ICB uses monoclonal antibodies that target the inhibitory proteins CTL antigen 4 (CTLA-4) or programmed death-1/programmed death-ligand 1 (PD-1/PD-L1) on T cells or cancer cells to unleash the immune response. However, response rates vary widely and predictive factors of response to ICB remain elusive. It has been suggested that PD-L1 expression, high tumor mutational burden (TMB) which is highly influenced by the epitopes displayed in the human leukocyte antigen (HLA) genes of a tumor, and the presence of CD8^+^ T cells are prognostic of clinical response to treatment with ICB (6).

The distinction between hot, altered (excluded and immunosuppressed) and cold tumors, based on the cytotoxic T cell landscape within a tumor, establishes the important role of the TME but only a thorough profiling of the TME can analyze the complexity of the tumors and provide dynamic information about the complex networks operating in the TME to guide clinical decisions (7, 8). Combining immune and genomic data has revealed six immune subtypes across 33 different cancer types including immune (macrophage or lymphocyte signatures, Th1:Th2 cell ratio, expression of immunomodulatory genes) and non-immune parameters (intratumoral heterogeneity, aneuploidy, neoantigen load, overall cell proliferation, and patients' prognosis) (9). It has been proposed that an integrative view of the multi-omics experimental platforms and computational power is required to identify signatures of immune response with improved predictive power (10).

It has been clearly established that CD8^+^ T cells are the ultimate effectors of tumor rejection and the strongest predictor of ICB response across tumor types. Significantly, the functional variability of tumor-infiltrating T cells can influence their cytotoxicity. Subsets with reactivation of dysfunctional CD8^+^, memory-like CD8^+^TCF7^+^, CD103^+^ tumor-resident CD8^+^, and Tcf1^+^PD-1^+^ CD8^+^ with stem-like properties T cells have shown durable responses. CD4^+^ T cell subpopulations that play a critical role in immunotherapy include CD4^+^ Th1 cells that generate functional CD8^+^ T cell responses, CD4^+^FoxP3^+^ regulatory T cells (T_reg_) generally associated with suppression of antitumor immune responses in several cancers although responses to CTLA-4 blockade have been shown, and CD4^+^FoxP3^−^PD-1^Hi^ (4PD-1^Hi^) T cells can indicate a negative prognosis when there is persistence after PD-1 blockade (6). Emerging factors associated with ICB response include B cells and tertiary lymphoid structures (11, 12). As for innate immune populations, BDCA-3^+^CLEC9A^+^ dendritic cells (DC) and XCL1-producing NK cells are linked to ICB response (13).

ICB is most efficacious in tumors with a high degree of T cell infiltration (hot tumors), such as melanomas and NSCLC. Alternative combinations include other checkpoint molecules, such as T cell immunoglobulin and mucin domain-3 (TIM-3), lymphocyte-activation gene-3 (LAG3), and T cell immunoglobulin and ITIM domain (TIGIT) in the case of T cell exhaustion; or co-stimulatory checkpoint proteins, including OX-40, CD28, and 4-1BB ligand receptor to enhance T cell expansion or effector functions. Preliminary results also suggest a potential role of microbiome modulation. On the other hand, immune cold tumors, including pancreatic and prostate cancers, are not well-infiltrated by immune cells. Therefore, research efforts have focused on making cold tumors hot by increasing immune infiltration and activity, such as vascular normalization, increasing the neoantigen burden, oncolytic therapy, vaccines, adoptive T cell therapy, T cell immunomodulators, and radiotherapy. Clinical strategies in immune-altered tumors have an impact on T cell trafficking, inhibition of hypoxia-associated pathways, and the immune suppressive microenvironment (14).

As more combinations of immunotherapeutic strategies reach the clinical arena, two clinical challenges become more relevant. Checkpoint disruption leads to a wide range of inflammatory toxicities grouped as immune-related adverse events (irAEs). The majority occur in barrier tissues (gastrointestinal or pulmonary mucosa, skin) or in endocrine glands. Although many are mild, they can carry considerable morbidity, lead to reduced treatment dosage and/or duration, and on occasions may be fatal (e.g., in patients with pre-existing autoimmunity) (15, 16). On the other hand, it has been suggested that irAEs could help select responders to ICB in bladder cancer (17). Secondly, some patients experience an acceleration of tumor growth kinetics with poor survival called hyperprogression which, at present, remains difficult to characterize (18, 19).

The composition of the TME is dynamic and evolves during ICB treatment. It has been suggested that the TME evolves differently between responders and non-responders. Of interest, stronger differences were found early on-treatment than before the ICB based on the differences in the densities of CD4^+^ or CD8^+^ T cells and the expression of PD-1/PD-L1 after two or three anti-PD-1 doses than at baseline (20, 21). Another interesting feature is that PD-1 blockade can induce clonal replacement preferentially of exhausted CD8^+^ T cells, meaning that T cells present at baseline may show reduced proliferation and that the response to ICB could be due to T cell clones that enter the tumor during the course of treatment (22).

Clinical relevance of distinctions in the immune contexture mainly based on the cytotoxic landscape of T cells in tumors has been established although the potential of analyzing dynamics and plasticity of TME networks will offer more powerful stratification systems between responders and non-responders.

### Interactions Within the TME

**Figure 1:**
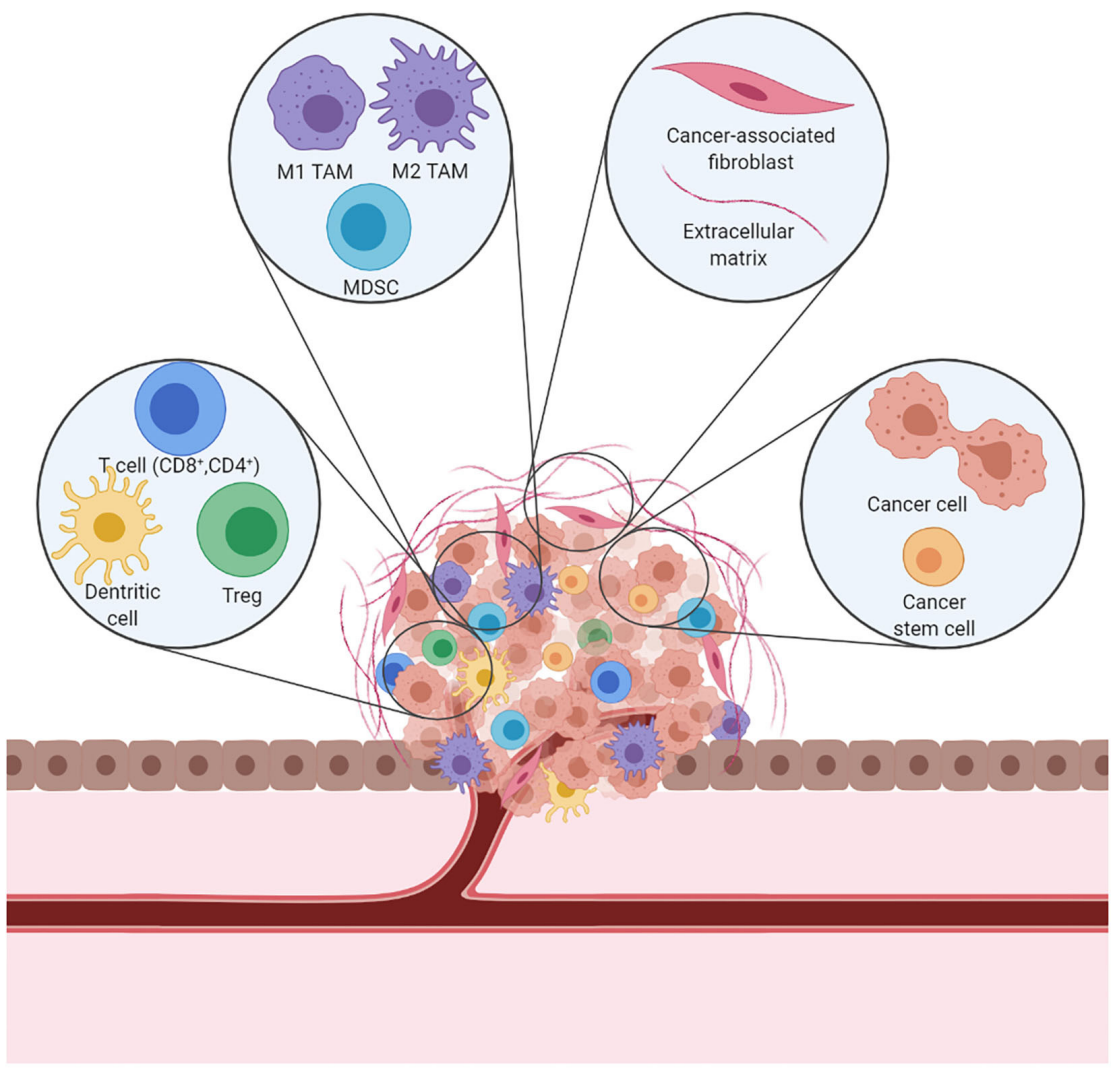
A schematic representation of the immunosuppressive TME. In a tumor, cancer cells coexist with immune cells, fibroblasts, and blood vessels to form the TME. Cancer cells can alter the microenvironment and promote cancer growth and dissemination.

Interactions between malignant and non-malignant cells create the TME (Figure 1). Non-malignant cells are usually highly dynamic and display tumor-promoting capabilities. Major non-malignant cell types found in the TME are immune cells, vasculature and lymphatic vessels, and fibroblasts. Cell communication is accomplished by a network of cytokines, chemokines, and diverse metabolites that reacts to changes in the physical and chemical characteristics of the tissue (23). Cancer treatment effects induce a variety of mechanisms which lead to T cell exclusion and avoidance of their cytotoxic function (24) that ultimately shift the balance of stromal cell phenotypes in the TME toward an immunosuppressive state. These pro-tumorigenic responses to therapy can induce local and/or systemic changes that underlie tumor recurrence and treatment resistance.

In a broad sense, the mechanisms leading to a pro-tumorigenic microenvironment can be grouped into three categories: immune cell regulation, metabolic reprogramming, and hypoxia (4). The biochemical and physical properties of the TME undergo substantial changes during tumor evolution and treatment determined by the increased demand for blood vessels to endure tumor growth, which requires an adequate supply of oxygen and nutrients delivered through the blood vasculature. The resulting abnormal vessels are leaky and compressed which can induce a dense stromal reaction and reduction of blood flow that promotes hypoperfusion. The TME then becomes hypoxic with enhanced potential for tumor progression in multiple ways. In this situation, hypoxia reduces immune cell activity and the TME acquires an immunosuppressive phenotype (25). Hence, better understanding and reprogramming of these components may greatly influence cancer outcome.

Clinically, this may significantly limit cancer treatment efficacy and represent a shift in our understanding of tumor progression and resistance. Major emphasis has been placed on advancing clinical applications that strengthen the effectiveness of immunotherapies, leading to rapid regulatory approval of ICB combined with targeted therapies and/or chemotherapy in large numbers of patients with cancer, facilitating their incorporation into clinical practice. However, in spite of the extensive use of surgery and radiation strategies in cancer, as a definitive strategy in early or moderately-advanced stages of cancer, as part of a multimodal strategy in advanced loco-regional disease and, more recently in selected cases of oligometastatic disease, there is very limited understanding of the biological changes in the TME induced by local treatments.

## Radioimmunotherapy Combinations

Radioimmunotherapy is an area of extensive research due to the potential immunomodulatory effects of ionizing radiation and has established a new paradigm in which radiation is as efficient as its capacity to elicit tumor-targeting immune responses (2).

Ionizing radiation is able to induce immunogenic cell death, a form of cell death that promotes a T cell-based immune response against antigens derived from dying cells, enhances antigen presentation, and activates cytotoxic T cells. Cytosolic DNA from dying cells function as neoantigens that are highly immunogenic. Radiation induces the release of danger signals, including calreticulin, high mobility group box 1 (HMGB1), and adenosine triphosphate (ATP), which are collectively known as damage-associated molecular patterns (DAMPs), and support the recruitment and maturation of antigen-presenting cells (APC), migrate to lymph nodes, and prime a cytotoxic T cell-dependent immune response.

Critical to the immunogenicity of radiotherapy is the fragmentation of nuclear DNA from the DNA damage response (DDR) of radiation, shuttled to the cytoplasm where it activates cyclic GMP-AMP synthase/stimulator of interferon genes (cGAS/STING) pathways and induces transcription of the IFN-stimulated genes. The cytoplasmic three-prime repair exonuclease 1 (Trex1), induced by radiation, is a negative regulator of this pathway. The release of IFN type I from APC supports antigen uptake by Batf3^+^ DC and cross-presentation of tumor antigens to CD8^+^ T cells. Activated CD8^+^ T cells are recruited to the irradiated tumor site by cytokines upregulated by radiation (CCL2, CXCL1, CXCL10, and CXCL16). In addition, radiation enhances the expression of major histocompatibility complex-I (MHC-I) antigens on cancer cells that favor antigen presentation (4).

### DNA Damage Response Following Radiation and Exposure of Neoantigens

Tumor cell-intrinsic events driven by DNA damage are central to the immunomodulatory effects of radiotherapy. Radiation-induced DNA damage alters gene transcription and modulates the expression of tumor neoantigens, resulting in activation of innate and/or adaptive antitumor immune response (6, 26, 27). The finding that a patient with metastatic, treatment refractory NSCLC who responded to ipilimumab plus radiotherapy was carrying a mutation in a *KPNA2* gene, upregulated in expression by radiation; tumor-specific T cell clones were developed in peripheral blood shortly after completion of radiotherapy and the first dose of ipilimumab to a metastatic site and remained elevated while the patient achieved a complete response in all of the non-irradiated lesions supports the hypothesis of *in situ* tumor vaccination (28).

Identification of genetic determinants of radiotherapeutic efficacy has remained elusive but a recent report identifies genetic *ATM* inactivation to be strongly associated with clinical benefit from radiotherapy. The identification of a radiosensitive phenotype across multiple cancer types inaugurates the possibility of further testing in prospective clinical trials and progress in personalized radiation strategies. For example, patients with metastatic tumors harboring a somatic *ATM* mutation may receive a reduced dose of radiation with the goal of reducing toxicity and maintaining tumor control (Pitter et al., accepted).

Defects in DDR have been exploited for drug development as radiosensitizers including poly(ADP-ribose) polymerase (PARP), checkpoint kinase 1 (CHK1), DNA-dependent protein kinase (DNA-PK), or the chaperone HSP90 inhibitors. Radiation damage in the context of defective DDR pathways generates micronuclei in cancer cells that activate cGAS/STING pathways and propagate an inflammatory response that can enhance radiation effects. Adding ICB to the immunomodulation induced by DDR inhibitors plus radiotherapy is a new area of clinical research that can provide additional insights into the immunomodulatory effects of radiation given that DDR inhibitors can enhance the immunostimulatory effects of radiation while ICB can target the immunosuppressive radiation effects (27).

### Central Role of Dendritic Cell Maturation in Radiation-Induced Immunological Response

DC are a sparsely distributed immunological component of the TME with high biological heterogeneity that play a central role in linking innate and adaptive immune responses. Therefore, DC are a key element in the immunostimulatory effect of radiotherapy. It has been recently reported that poorly radioimmunogenic murine tumors fail to activate DC following treatment, and that it could be successfully reverted with an exogenous adjuvant, resulting in tumor cures (29). Therefore, it could be hypothesized that in patients with a poor TME, the combination of radiation with adjuvants that promote DC maturation or target the immunosuppressive TME can improve tumor control.

Toll-like receptors (TLR) signaling pathways activate innate immunity and regulate adaptive immune responses. Preclinical evidence suggests that TLR-agonists targeting TLR3, TLR 7/8 or TLR9 in combination with radiotherapy can enhance antitumor immunity with long-term tumor control. Mechanistically, TLR can enhance DC-mediated cross-presentation and activation of T cells. Novel formulations of TLR agonists with reduced toxicity and precise and image-guided radiation techniques are favorable aspects for this strategy (30, 31).

### Addressing the Evasive Objective of Durable Responses of Radiation-Immunotherapy Combinations

Studies on resistance to ICB reveal a complex and rapidly evolving network of mechanisms of immune resistance specific to each host and tumor (32). The absence of biomarkers that identify the different types of resistance obliges the use of empirical approaches to target them.

The immunogenicity of radiation has been approached with two different strategies, one that emphasizes the local interaction of radiotherapy and the immune system where the majority of clinical knowledge has been accumulated, and a second strategy where focal radiation elicits systemic disease control (abscopal effect) known as *in situ* tumor vaccination that has attracted a lot of attention. The basis for combining ICB with radiotherapy stems from the fact that radiation upregulates PD-L1, which leads to CD8^+^ T cell exhaustion. In addition, many tumors devoid of T cells at baseline (and secondary lack of PD-L1 expression on effector T cells) could benefit from the radiation-induced increase in PD-L1 and the combination (33). In the case of CTLA-4, upon radiation, it is recruited to the membrane of activated T cells and binds to the ligands CD80 and CD86, expressed on DC and other APC, thereby attenuating T cell activation (34).

Tumor burden has been regarded as a surrogate for ICB effectivity based on clinical observations that adjuvant ipilimumab in resected stage III melanomas obtains major benefits in recurrence-free survival and overall survival (48.3 and 65.4% at 5 years, respectively) (35), and locally advanced NSCLC treated with definitive chemoradiation followed by adjuvant durvalumab in the PACIFIC trial with an impressive prolongation of time to death or distant metastasis from 16.2 to 28.3 months and a favorable toxicity profile (36). Moreover, in patients that do respond to ICB, failure frequently occurs in sites of previous disease, with 60% of failures in anti-PD-1/PD-L1 treated NSCLC and 39% of failures in anti-PD-1 treated melanoma (37, 38). Although it is not specific criteria, the best outcome with ablative radiation in oligometastatic clinical trials has been obtained in patients with low tumor burden and as local consolidation (39, 40).

While the majority of clinical studies have targeted a single metastatic site, abscopal responses are relatively rare, and mainly in melanoma and NSCLC (41). Improved outcomes have been obtained in several phase 2 clinical trials using local consolidation with ablative doses of radiation in the oligometastatic setting (39, 40, 42) while ongoing phase 3 trials are investigating whether this approach may lead to improve overall survival in a subset of patients with limited metastatic disease (NCT02417662, NCT03137771, NCT02364557, NCT03862911, and NCT03721341).

It has been recently reported that tumor-resident CD8^+^ T cells play a significant role in mediating the immune effects of radiotherapy. Even if proliferation decreases after radiation, their functionality, measured as production of IFN-γ, augments, and mediates the early antitumoral effect of local SBRT doses. Nonetheless, as newly infiltrating CD8^+^ T cells play a key role in antitumor immunity, that may also be the case with radiation-induced immunogenicity (43). If radiation could increase the population of pro-immunogenic T cell subtypes within the local TME, it would enhance the response to ICB. This hypothesis raises the possibility that targeting multiple metastatic sites with SBRT to achieve complete cytoreduction in the metastatic setting may become clinically relevant (44). Moreover, the irradiation of each visible metastasis addresses the challenge of heterogeneity by attempting to convert each target into an *in situ* vaccine (45). Clinical support data comes from a phase 2 clinical trial in NSCLC with up to 4 metastatic sites (93% had <2 metastases), which underwent locally ablative treatment with metastasectomy or multi-site SBRT followed by pembrolizumab, with median survival of 19.1 months (vs. 6.6 months in historical controls) and favorable toxicity profile (46).

Research has been very controversial with variations in dose or fraction for radiation delivery in the metastatic setting, where the role of treatment parameters such as duration (more or <7 days), fraction size (1.8–3 to 8–30 Gy) and scheduling (single or multiple fractions) are largely unknown. While a short course (1–5 fractions) of high dose radiation can be safely administered and is able to elicit an immunogenic response that can benefit from the addition of ICB, the predominance of the immunosuppressive effects of radiation may limit the effectiveness of ablative doses of radiation, especially if single fractions are used (47, 48). Nonetheless, the immune context of the tumor type or even the metastatic organ may require a different dose and/or fractionation to elicit an immunogenic response. This possibility offers the potential to reduce the dose and volumes of radiation and still prove efficacious. In the PACIFIC trial (36), immunotherapy was administered sequentially (i.e., following chemoradiation) with a good toxicity profile but data on toxicity of concomitant radiation and immunotherapy in the clinical setting is scarce. Yet the biological context remains to be proven that would favor multiple rounds of high-end ablative dose schedules in oligometastatic patient as advocated by some groups (49). Another concept of potential clinical relevance that has been put forward is the possibility that the immunomulatory effect of low-dose radiation for stromal modulation could favor T cell infiltration and enhance the immune response (47, 50).

The next generation of clinical trials addressing radiotherapy-immunotherapy combinations will have to include immunological read-outs with proper endpoints for immune monitoring as well as the identification of immune biomarkers that optimize the selection of treatment strategies (31, 51).

## Clinical Implications of Technological Developments

Surgery and radiation remain strong curative modalities for treatment of established solid tumors but treatment failure continues to be a significant problem. The best established role of surgical oncology is the complete removal of the tumor, with an additional strong foundation to question the elective treatment of uninvolved regional lymph nodes in a large variety of tumor types and resection of metastatic disease which is increasingly offered to selected patients with indolent oligometastatic disease (52). Critical to all of them is securing negative surgical margins.

Less invasive technologies and advances in imaging leading to minimally invasive and robot-assisted surgeries are revolutionizing surgical care (5). Likewise, advanced image guidance and motion management strategies are shaping new therapeutic radiation strategies enabling the safe administration of ablative doses of radiation (2). Advanced imaging is fundamental and uniquely placed to serve both margin negativity rates and future radiation strategies.

Surgical margin positivity rate (cancer cells at the edge of tumor resection) has not significantly improved in recent decades and when it does occur prognosis is significantly affected in many tumor types. Margin positivity rates across all types of cancer range from 15 to 60% (53). A recent report on positive surgical margins in the ten most common solid cancers has identified oral cavity cancer with the highest rate with up to 25% of cases, no change over time, with significant effects on tumor relapse and overall survival. For advanced disease, the rates ranged between 20.9% (breast) and 65.5% (prostate) with related worse outcome in seven tumor types (54). Although not a true resistance type, we propose the term “margin-missing” effect to characterize this situation which leads to treatment failure and resistance.

Fluorescence-guided surgery, which allows intraoperative visualization of tumors, is an evolving image-guided surgical strategy to help differentiate tumor cells from normal surrounding tissues in real time. Near-infrared fluorescence imaging has a higher tumor to background ratio, high tissue penetration (5–10 mm), and little interference from intrinsic fluorescence. Indocyanine green is the most widely used probe in fluorescence-guided surgery although tumor detectability is not very good and optical technology is still evolving (55).

More than 50% of patients with cancer receive radiotherapy, which defines its leading role in cancer management, in particular for several locally advanced solid tumors. The latest developments in radiotherapy have swiftly enabled local dose escalation making it possible to deliver high doses of radiation with incredibly high anatomical precision and reduced risk of long-term adverse effects. As a consequence, relevant clinical benefit has been achieved in a variety of cancer types such as prostate, gynecologic, breast, head and neck cancers, and brain and lung metastases (2). However, no significant advance has occurred in the past 30 years in the development of strategies that enhance radiation effects. On the other hand, due to the recognition that the immune system can strongly contribute to therapeutic responses to radiation, radioimmunotherapy has become an intensive area of research.

The current challenge in near-infrared fluorescence-guided surgery is to design probes with high selectivity for tumors and clear visualization, referred to as smart probes, which are only activated at the tumor site (turn-on probes). There was a recent report about the design, synthesis, and characterization of three novel polymeric turn-on nanoprobes that are activated at the tumor site by cysteine cathepsins (highly expressed in multiple tumor types) showing a stable and well-defined signal from the tumor during the whole surgical procedure in orthotopic breast cancer and melanoma models resulting in less tumor recurrence and prolonged survival compared with standard commercial probes (56). This is a significant lead toward real-time image-guided tumor margin assessment during surgical oncology.

Emerging approaches seek to integrate analytical tools with optical technology to help improve the decision-making of fluorescence-guided surgery to reduce margin positivity rates. For example, combinations of fluorescence-guided surgery have been made with mass spectrometry (57), Raman spectroscopy (58), and hyper spectral imaging (59).

The most clinically advanced nanoprobe is LUM015 (a pegylated cathepsin-activatable probe) which is undergoing eight clinical trials, including a pivotal phase 3 study (60). The phase 3 trial is a multicenter study with the primary objective of assessing the ability of LUM015 and LUM fluorescence-guided surgery system to detect residual tumors in 250 breast cancer patients undergoing lumpectomy (NCT03686215).

## Clinical Implications of the Immunosuppressive Environment

It has been traditionally assumed that recurrent tumors arise from transformed neoplastic clones that are more resistant to oncological therapies, however, an early experience challenged this view and hypothesized that primary and recurrent tumors of equal size did have different microenvironments that explained their response to therapies. The study found that while small primary tumors had a healthy population of antitumor effector CD8^+^ T lymphocytes, recurrent tumors had an immunosuppressive condition consisting in expanded populations of tumor-associated macrophages (TAMs), T_reg_ cells, and pro-tumoral cytokines that inhibited cytotoxic CD8^+^ T lymphocytes. These changes were also identified in regional draining lymph nodes. Disruption of these immunosuppressive pathways restored the efficacy of the tumor vaccine in recurrent tumors, as if they were primary tumors (61).

Research in preclinical models has shown that a syringeable immunomodulatory multidomain nanogel (iGel) containing gemcitabine, imiquimod, and clodronate locally applied as a postsurgical treatment is able to deplete immunosuppressive cells from the TME (myeloid-derived suppressor cells (MDSCs), M2 macrophages, and T_reg_ cells), increase immunogenicity, and induce immunogenic cell death. Indeed, it generates systemic antitumor immunity and a memory T cell that significantly inhibits tumor recurrence and lung metastases. Reprogramming the immunosuppressive TME also converts tumors not responding to ICB to responding ones (62). This platform may serve to reshape immunosuppressive TME and synergize with other therapies.

Recent clinical data in melanoma and NSCLC have shown that response to ICB in individual patients with metastasis vary depending on the anatomical location of the metastasis, untangling the importance of the local TME in antitumor immunity. Of interest, tissue specific response to immune checkpoint inhibition depends on the cancer type, which implies that responsive and non-responsive sites are different among patients with NSCLC or melanoma (63, 64). These heterogeneous responses are an evident clinical problem, since patients with responses to ICB in all lesions survive longer than those with response in some of the lesions (65). Potential mechanisms include myeloid cell exclusion and alteration of T cell activation in response to tumor growth and local factors, but this will require unraveling a very complex network of interactions for differential responsiveness across different tissue sites of tumor deposits.

### Regulatory T Cells

T_reg_ cells are a small subset of circulating CD4^+^ T cells with potent suppressive functions with a central role in regulating immune responses and maintaining self-tolerance although they also impede antitumor immunity. In contrast with circulating T_reg_ cells, intratumoral T_reg_ cells maintain an active configuration, suggesting that antigen stimulation may play an important role in the activation and accumulation of T_reg_ cells in the TME. The immunosuppression mediated by T_reg_ cells is mainly mediated by the release of anti-inflammatory cytokines including IL-10 and transforming growth factor β (TGFβ), facilitating proliferation of CD4^+^ T cells to T_reg_ cells, while suppressing proliferation to CD8^+^ T cells and NK cells. In addition, T_reg_ cells can also reprogram macrophages to the M2 phenotype (via IL-4, IL-10, and IL-13) and favor MDSCs infiltration (via IL-10 and IL-35) (66).

Immunological cell death induced by radiation upregulates or releases DAMPs, including ATP, with further recruitment and activation of DC to initiate the antitumor immune response but ATP is rapidly catabolized in the TME into adenosine by the enzymes CD39 and CD73. Local accumulation of extracellular adenosine suppresses DC and CD8^+^ T cells and promotes proliferation of T_reg_ cells, increases the expression of CTLA-4 and adenosine receptor A2 (A2AR) on T_reg_ cells, and enhances the polarization of macrophages to the M2 phenotype. Radiation can also induce conversion of ATP to adenosine through the induction of reactive oxygen species (ROS) and TGFβ. Thus, targeting of A2AR, CD73, and TGFβ may reduce resistance to immunotherapy in the radiotherapy setting (33). Blockade of CD73 plus radiotherapy restored radiation-induced DC infiltration of tumors in a poor immunogenic setting, and the addition of CTLA-4 blockade improved control of non-irradiated lung metastases in murine models. These findings set the stage for clinical testing CD73 in patients who carry cGAS/STING tumors or show upregulation of soluble CD73 following radiotherapy to determine if CD73 blockade can enhance responses to ICB (67).

T_reg_ cells also express PD-1 at a low level in the blood and at a high level in tumors, promoting the suppressive activity of PD-1-expressing T_reg_ cells upon antibody-mediated PD-1 blockade (68). Recently reported, ~10% of cancer patients treated with anti-PD-1 antibody develop hyperprogressive disease, characterized by rapid cancer progression. T_reg_-specific depletion prior to, or combined with, an anti-PD-1 antibody may prevent hyperprogressive disease and enhance the effectiveness of anti-PD-1 therapy (69).

### Macrophages

TAMs account for the largest fraction of the myeloid infiltrate in the majority of solid tumors. The tumor-associated macrophage compartment is highly dynamic in time (during tumor progression and response to treatment) and space (at different tumor sites) through an extensive remodeling of energy metabolism. In addition, the tumor-associated macrophage compartment is highly heterogeneous both within and across tumors in response to environmental changes ranging from a pro-inflammatory (M1) to an anti-inflammatory (M2) state. However, the M1/M2 phenotypes represent the extremes of a continuum and the plasticity of these cells makes therapeutic targeting challenging. Solid experimental evidence informs that the crosstalk between TAMs and the immune cells facilitates an immunosuppressive environment by supporting angiogenesis and extracellular matrix (ECM) remodeling, promoting active recruitment of T_reg_ cells, and expression of PD-L1, paving the way for metastatic development (70). M2 polarization is mostly mediated by growth factors and cytokines secreted by cancer cells that reach M2 cells via exosomes (71). Intriguingly, ontogeny can influence the functional profile of TAMs, i.e., tissue-resident vs. circulating macrophages, such that they can have opposing functions depending on the tumor type (72). Based on these findings, it has been speculated that macrophage origins may be important in determining the permissiveness of an organ to metastatic growth.

Preliminary studies have evaluated the influence of radiation in macrophage polarization. Macrophages are highly radioresistant due to high production of anti-oxidative molecules such as manganese superoxide dismutase by a mechanism depending on tumor necrosis factor α (TNFα) signaling and nuclear factor-κβ (NFκβ) activation (73). Early studies established that radiation exposure recruited bone marrow-derived CD11b^+^ monocytes/macrophages to irradiated sites (74, 75) and related it to the transcription factor hypoxia-inducible factor-1α (HIF-1α) and effectors stromal cell-derived factor-1 (SDF-1) and C-X-C chemokine receptor type 4 (CXCR4) (76, 77). Therapy can polarize macrophages to the M2 phenotype with very high levels of proangiogenic molecules through the treatment-induced expression of colony stimulating factor 1 (CSF-1), the ligand for the colony stimulating factor 1 receptor (CSF-1R) on macrophages, which can be prevented by CSF-1R antagonists and enhance radiation effects (78, 79).

**Figure 2:**
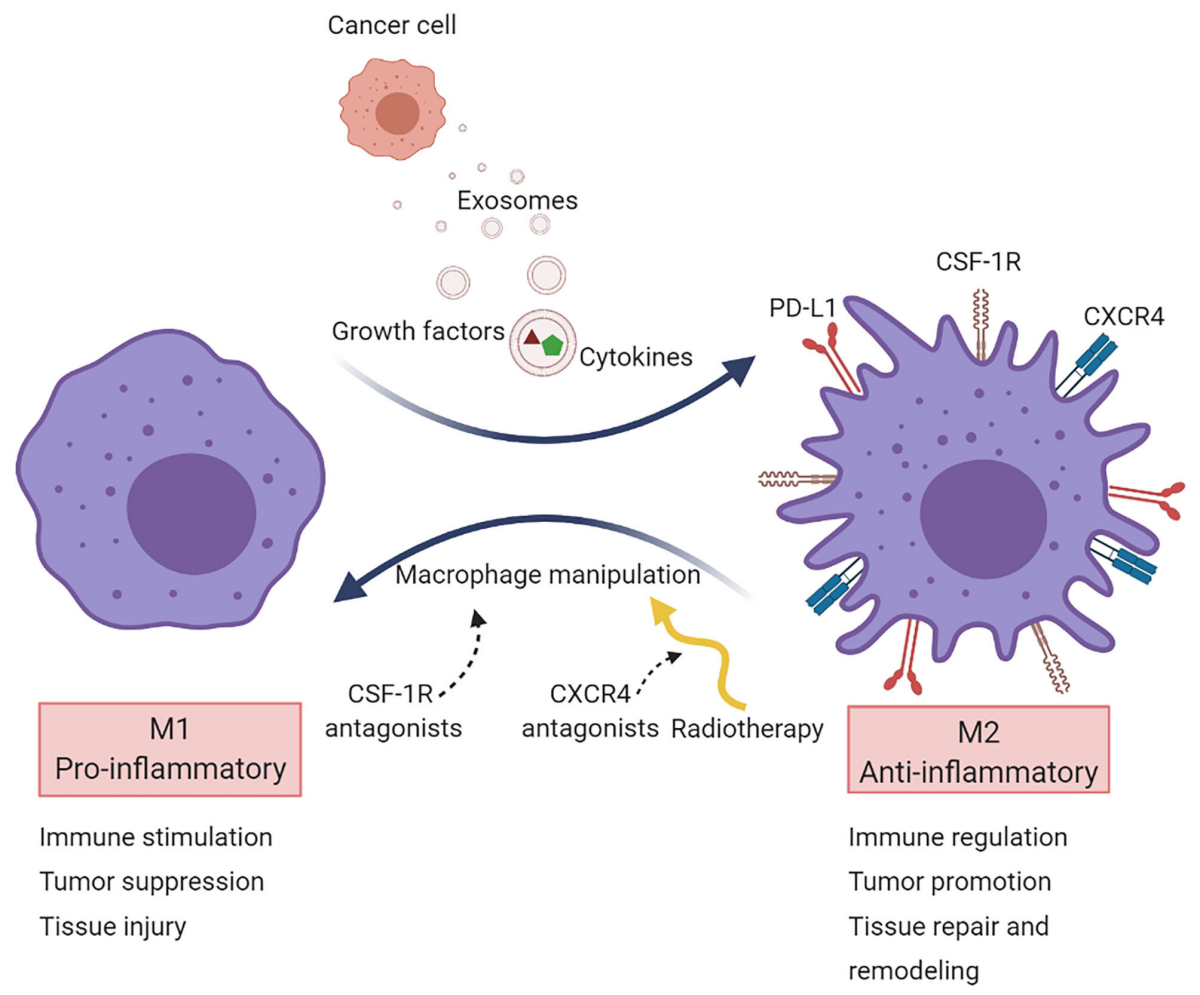
Macrophage targeting in cancer. Macrophages are primarily recruited to tumors to acquire a pro-tumorigenic phenotype (M2 state). Several strategies target TAMs aiming to reprogram them into a pro-inflammatory phenotype (M1 state). Most macrophage-targeted therapies are focused on CSF-1R inhibitors. Another approach is via CXCR4 blockade, which acts on vasculogenesis and has been tested in the clinical setting after radiotherapy in glioblastoma.

Ongoing research efforts are directed toward the alteration of the macrophage phenotype to attenuate immunosuppression and improve antitumor immunity (80). Current approaches aim to shift M2 cells to M1 by targeting secreted immunosuppressive factors released by cancer cells and cells in the TME (Figure 2).

Preclinical studies suggest that macrophage manipulation to avoid recruitment or prevent M2 polarization produce a significant enhancement of the radiation effect irregardless of the tumor model [reviewed in (81)]. The increase in radiosensitivity with this strategy has been attributed to blockade of vasculogenesis. If angiogenesis supports the formation of tumor blood vessels from the sprouting of local vessels, tumors can also develop or repair blood vessels from circulating proangiogenic cells mainly from the bone marrow, which is known as vasculogenesis (82). This effect could be exploited in radiation treatments, namely if the increase in hypoxia that occurs at the end of radiation through recruitment of circulating proangiogenic cells to rescue damaged tumor vasculature and promote tumor recurrence can be reversed. A first-in-human clinical trial of glioblastoma examined the effects of CXCR4 blockade through a 4-week continuous infusion of plerixafor, a small molecule CXCR4 inhibitor, at the end of irradiation in newly diagnosed glioblastoma and showed high efficacy and local control with an excellent median survival time of 21.3 months. Unexpectedly, a high proportion of patients had out-of-field recurrences with local tumor control which deserves further evaluation (83).

A relevant aspect that remains unanswered is whether the effect of blocking the CXCR4 pathway could be more pronounced with ablative doses of radiation which seems likely since greater vascular damage would be expected. Furthermore, it is of interest to know if CXCR4 blockade can enhance tumor immunity. Very limited information suggests that T cell exclusion from cancer cell deposits secondary to SDF-1 could be overcome by inhibiting the CXCR4 axis, improving the effect of checkpoint inhibitors or stroma normalizing strategies in pancreatic cancer (84, 85) and triple-negative breast cancer (86) models.

### Pre-metastatic Niche and Exosomes

In addition to TAMs, radiation also recruits MDSCs in the irradiated tumors by tumor-secreted factors like SDF-1. MDSCs encompass a heterogeneous population of polymorphonuclear MDSCs and monocytic MDSCs which inhibit the activity of CD8^+^ T cells. Moreover, MDSCs play a prominent role in the establishment of the pre-metastatic niche, promote angiogenesis and facilitate the development of metastasis (87).

**Figure 3:**
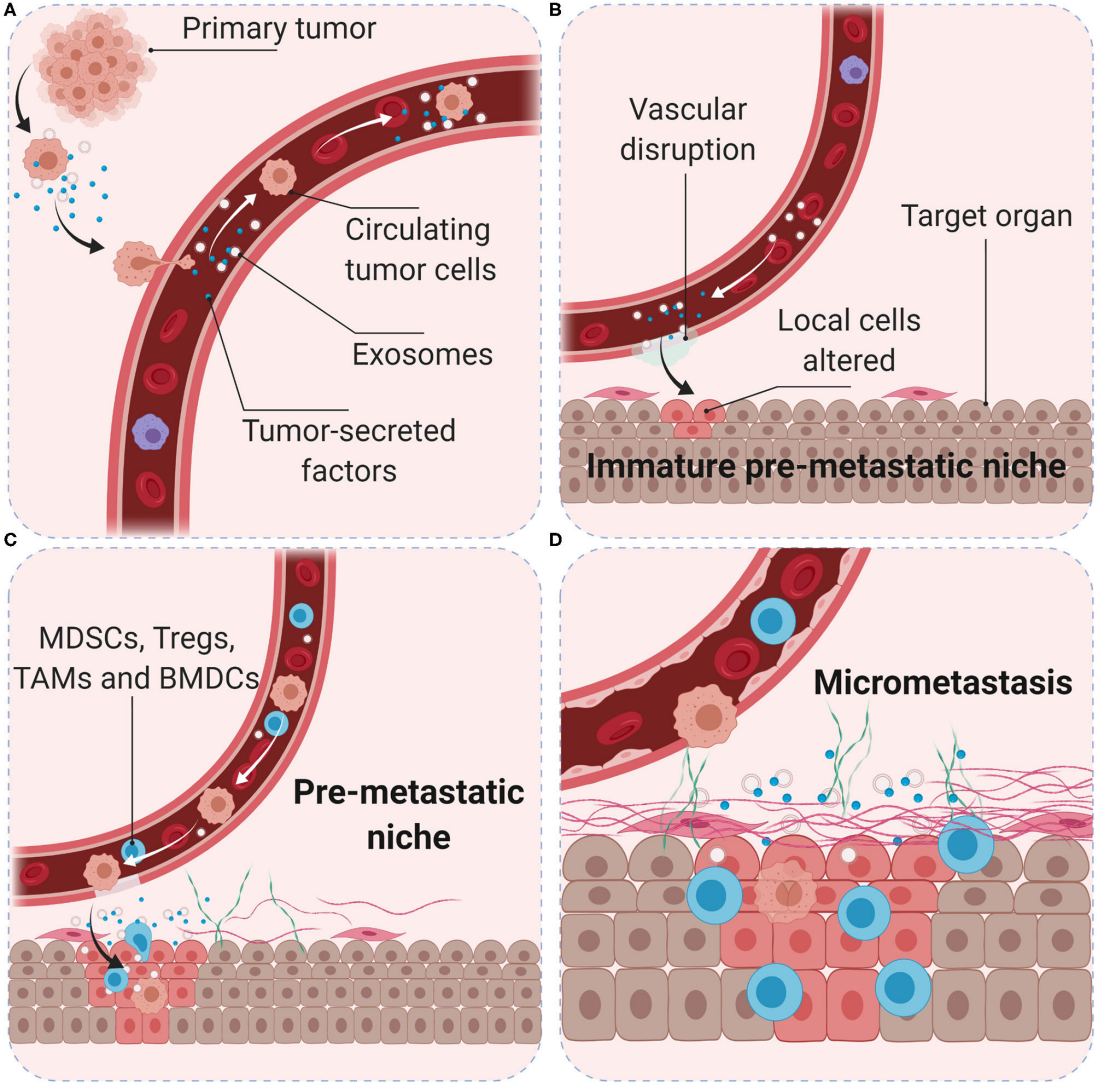
Role of the pre-metastatic niche in cancer metastasis. Primary tumor cells produce soluble factors and exosomes **(A)** to trigger the formation of an immature pre-metastatic niche in the target organ **(B)**. Primary tumor conditions (hypoxia, acidity, and interstitial pressure) promote tumor cell migration into the blood vessels. Tumor-secreted factors and exosomes mobilize bone marrow-derived cells (such as CD11b+ myeloid cells) and suppressive immune cells (such as MDSCs, T_reg_, and TAMs) to target organs **(C)**. Interactions with local stroma, hypoxia and active ECM remodeling may create a niche with suitable microenvironment conditions for tumor cell colonization **(D)**. Surgery, inflammation, and immunosuppression may increase the number and survival of circulating tumor cells and favor the development of metastasis.

Tumors induce the formation of microenvironments in distant sites that support future metastatic tumor growth before their arrival at these sites, known as pre-metastatic niches. Tumor-secreted factors and tumor-shed extracellular vesicles promote a sequence of events that start with vascular leakiness, and are followed by alteration of local cells in the TME, recruitment of MDSCs, and finally attraction of circulating tumor cells (88). Following seeding in a secondary organ, cancer cells interact with their environment to create the metastatic niche. The microenvironment in pre-metastatic niches is immunosuppressive and MDSCs are the main cellular component, however, migration of MDSCs into pre-metastatic niches and subsequent activation is not well-characterized. More than 100 different immunosuppressive tumor-secreted proteins have been identified (89). Fibronectin accumulates and anchors to collagen in the ECM to facilitate the adherence of circulating tumor cells through high affinity of membrane integrins (90). The vascular changes allow for uptake of tumor-secreted exosomes by cancer-associated fibroblasts (CAFs) in the local stroma, which contributes to the formation of a tumor-associated desmoplastic stroma, characteristic of many carcinomas (91) (Figure 3). Exosomes are extracellular vesicles released by exocytosis and essential to intercellular communication. They can contain genetic material, proteins, and lipids; they can be found in all body fluids and are considered to be major drivers of pre-metastatic niche formation (92). Measurement of exosomal microRNA has been shown to accurately reflect tumor progression in several cancer types (93, 94) as well as dropping levels of exosomal microRNA after surgery indicate that the resected tumor was the main source of exosomal release (95). However, in animal models of abdominal cancer surgery can induce increased levels of ROS, that may downregulate tight junctions in the endothelium and peritoneum, form intercellular gaps and expose the underlying ECM; which can promote integrin-binding of circulating tumor cells during surgery, and result in an excess of liver metastases in a colorectal cancer model (96).

Research in animal models of breast cancer known to produce immunosuppressive MDSCs in the spleen and lungs, has shown that surgical resection of the primary tumor decreased levels of MDSCs in the spleen but persisted in the lungs for 2 weeks after resection, indicative of a pro-metastatic environment. Post-surgical treatment with gemcitabine depleted lung MDSCs and decreased posterior metastatic disease (97).

Accumulating evidence indicates that exposure to radiation induces the release of exosomes (98–100) that could contribute to radioresistance but additional mechanistic understanding to define potential interventions is lacking. The potential role of exosomes has also been explored as biomarkers of disease outcome in head and neck cancer patients treated with cetuximab, radiation, and ipilimumab; exosomes were isolated from plasma and the molecular cargo contents (derived from T_reg_ cells) could separate patients who remained free 2 years after treatment from those who did not (101).

An important aspect required to characterize extracellular vesicles is the development of highly specific detection techniques. Since the distribution of extracellular vesicles in the TME depends on the cellular function, it is highly necessary to visualize them in freshly resected tissues. There was a recent report about an intraoperative optical imaging system that was able to provide rich details and molecular contrast thanks to a label-free multimodal nonlinear optical technology in human breast cancer showing good correlation with stained histological slides. The enriched areas with extracellular vesicles in the microenvironment correlated with macroscopic tissue deposits as well as increasing distance from tumor to margin (102).

A recent publication has shown that after surgical removal of resected primary lung, breast, and esophageal cancer, low-dose adjuvant therapy with epigenetic therapy can disable the pre-metastatic niche and inhibit the formation of lung metastases by avoiding the trafficking of MDSCs and promoting their differentiation into a macrophage-like phenotype (103). These preclinical findings represent a novel paradigm to be tested in clinical trials.

### Cancer-Associated Fibroblasts

**Figure 4:**
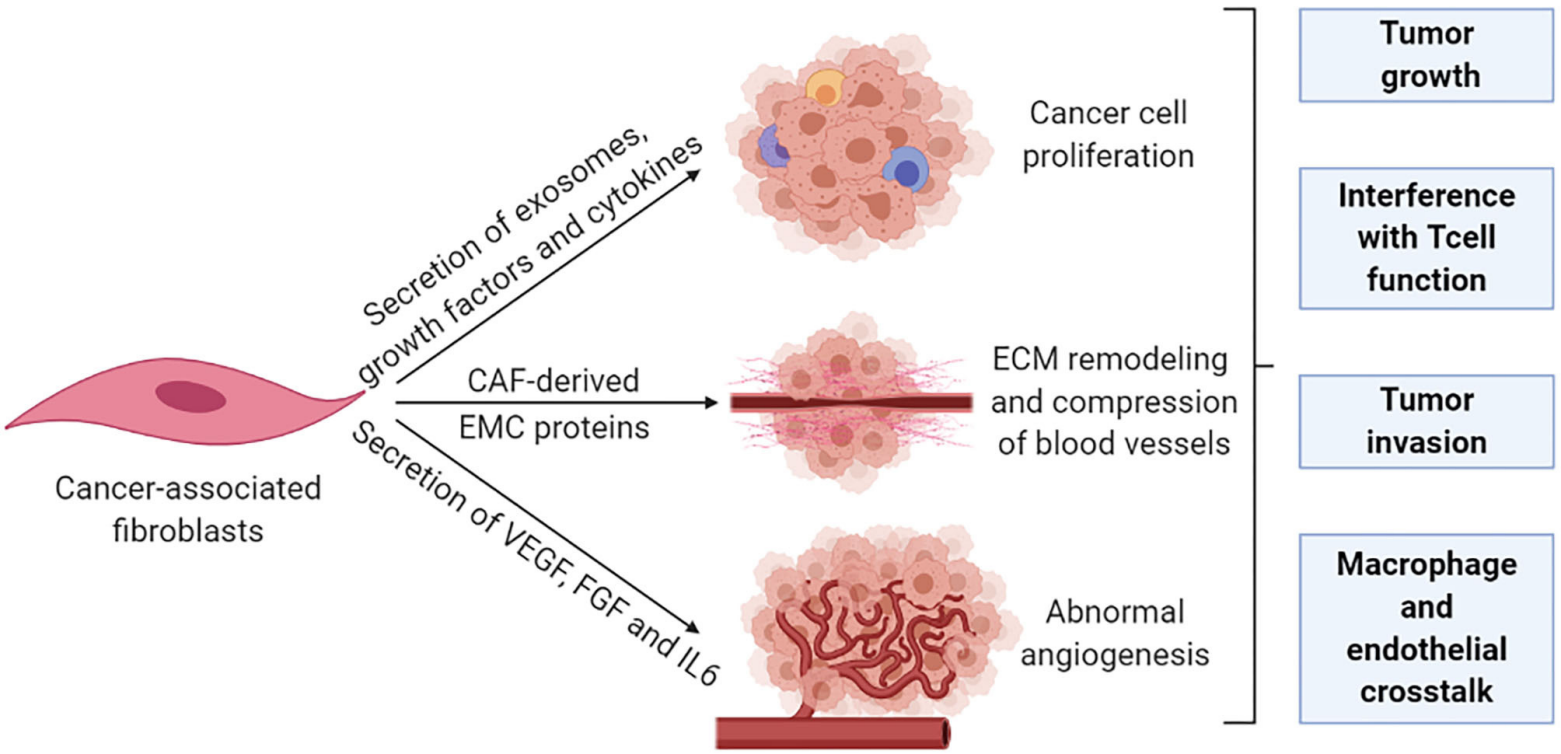
Cancer-associated fibroblasts remodel the tumor stroma. The pro-tumorigenic functions of CAFs are generally associated with their highly secretory activity. Secretory functions and matrix remodeling contribute to tumor invasion and angiogenesis. In addition, secreted soluble factors also contribute to immune reprogramming and tumor growth. Metabolic remodeling by CAFs supports an immunosuppressive microenvironment and promotes tumor growth.

Fibroblasts, the major cell type in the TME, are critical determinants of cellular crosstalk (104). CAFs, a subpopulation of activated fibroblasts, are difficult to identify and in practice, are described as any mesenchymal cell that lacks lineage markers for epithelial cells, endothelial cells, and leukocytes. CAFs are proliferative, migratory, and highly secretory cells that promote extensive tissue remodeling which influences the physical and chemical properties of the tumor and increases the ECM stiffness, which promotes malignancy in experimental models. An extensive range of functions have also been attributed to CAFs, including secretion of growth factors, cytokines, and exosomes that promote tumor growth and alter treatment responses. The principal effect of CAFs is considered to be immunosuppressive with IL-6, SDF-1, and TGFβ as well-established mediators (105). These CAFs contribute to a rigid matrix that creates a physical barrier that leads to vessel compression and reduces diffusion of therapeutic agents to cancer cells which are particularly relevant for colorectal and pancreatic cancer (106, 107). CAFs are also effective in the remodeling of the tumor vasculature through the secretion of vascular endothelial growth factor (VEGF), fibroblast growth factor, and IL-6 to enhance angiogenesis (108, 109) (Figure 4).

Emerging evidence implicates CAFs in immune escape and resistance to immunotherapy but not all subpopulations seem to have the same functions. A comprehensive identification of specific subsets of CAFs and their function is needed to become a viable targeting option (110). Currently, several preclinical strategies that target specific subsets of CAFs are under development (109).

Two promising strategies are normalization of activated CAFs, which intends to revert the activated state into a quiescent state or to induce them to acquire tumor-suppressor phenotypes (111), and targeting CAF-derived ECM proteins, either their production or degradation to alleviate the ECM stiffness (109). Reprogramming of CAFs to enhance immune responses, normalizing their ECM, is being investigated through the addition of vitamin D analogs (known to convert them into a quiescent state) to ICB in pancreatic cancer, and through TGFβ blockade combined with immune checkpoint inhibition in multiple tumor types (111).

## Clinical Implications of Metabolic Remodeling

Metabolic crosstalk across all cellular compartments is responsible for homeostasis and evolution of the TME. All cells of the TME, both malignant and non-malignant, compete for nutrients and oxygen, which are generally limited, especially in a stiffened and poorly vascularized TME, or secondary to the accumulation of the excessive production of metabolites by cancer cells. Additional aspects that influence how the TME reacts include immune-related substances released by cancer and/or immune cells, mechanical forces in the ECM, and reactions to treatment (112).

Although the metabolic pathways are shared between cellular compartments of the TME, the singularity of the reaction of stromal cells to energy demands is crucial. TAMs and CAFs are recruited to the tumor bed and activated in response to different stressful situations, such as limited nutrient disposal, hypoxia, and oxidative stress, attracted by cytokines such as TGFβ and CXCL2 or ROS from cancer cells [reviewed in (113)]. In such complex interactions, metabolites can serve different roles such as being a source of energy or communicate signals between different cellular compartments, and metabolism byproducts can favor an immunosuppressive phenotype. CAFs can rapidly adapt to these poor conditions through glycolysis and fatty acid oxidation in mitochondria. This increased consumption of glucose is coupled with extensive lactate secretion, which acidifies the TME and facilitates the activation of TAMs (114). The result of this swift metabolic adaptation of CAFs is the secretion of ECM-remodeling enzymes that promote fibrosis and further limit the availability of nutrients and oxygen, establishing a dynamic circuit in which lactate accumulation, glucose deprivation, and hypoxic conditions stimulate the recruitment and activation of additional stromal cells (113). Hypoxia supports the stabilization of the transcription factor HIF-1α to foster glycolysis. In this setting, HIF-1α also mediates CAF-secretion of proangiogenic factors such as VEGF, and hypoxia contributes to tumor progression by stimulating CAFs to secrete immunomodulatory molecules, growth factors, antioxidants, and ECM-remodeling enzymes. Taken together, the response of CAFs under poor nutritional conditions promotes tumor progression through engagement of endothelial cells. In addition, altered metabolism of cancer cells can create a gradient of metabolites around the tumor that can signal the distance to blood vessels and tailor the secretion of VEGF to match the tumor spatial organization and optimize the angiogenic response (115), and the metabolic switch in the TME may add to the disrupted immune cell metabolism (80).

Amino acids synthesize nucleotides and are also intermediate metabolites that contribute to other bioenergetic pathways. Glutamine is an abundant nutrient that provides carbon and nitrogen for pathways that contribute to energy formation, redox, homeostasis, macromolecular synthesis and signaling for cancer-cell growth of particular relevance in hypoxic conditions (116).

Endothelial cells form the lining of blood vessels and lymphatics and require glycolysis for proliferation and migration during angiogenesis. As the tumor grows, new vessels are required to supply the tumor with nutrients and oxygen and the endothelial cells increase their synthetic and energetic demands. Sprouting, the formation of new vessels, is a well-known mechanism in the angiogenic process and an area of intensive research. Although endothelial metabolism has been mainly described as glycolytic, recent findings suggest that mitochondrial oxidative phosphorylation is also required for endothelial cell proliferation during angiogenesis (117).

The interplay between metabolic remodeling and immune regulation in cancer is an active area of investigation. Preclinical models in glioblastoma have identified that tryptophan and adenosine metabolism result in accumulation of T_reg_ cells and M2 macrophages, contributing to an immuno-suppressive phenotype. Future studies will need to define the role of the intermediary metabolites of these pathways to determine their therapeutic function (118).

Preclinical results with the prodrug JHU083, a glutamine antagonist that targets glutaminase and a broad range of glutamine-requiring enzymes, provide a strong and differentiated metabolic response in which cancer cells stop growth, through depletion of glutamine pathways and impairment of glucose uptake, and in addition stimulates T cell functionality, even with persistent antitumor memory (119). Disengaging the metabolism of cancer cells and that of T cells is an evolving therapeutic concept.

A link has been recently proposed between ECM stiffness and metabolic transformation that facilitates tumor progression. It was found that through metabolic crosstalk between CAFs and cancer cells, aspartate secreted by CAFs maintains cancer cell proliferation while glutamate secreted by cancer cells balances the redox state of CAFs to promote ECM remodeling. This amino acid exchange among glutamate and aspartate offers new targeting options for both stromal and cancer cells (120).

## Clinical Implications of Angiogenic Responses

An abnormal vasculature is a paramount characteristic of solid tumors, with suboptimal function resulting from a leaky and immature vessel network (via overexpression of proangiogenic molecules such as VEGF), and compression of these anomalous vessels by physical forces (via TME cells and the ECM molecules they produce) (121). The resulting hypoxia enforces the stimulation of immune checkpoints and infiltration of immunosuppressive cells in the TME (122). Specifically, hypoxia up-regulates immune checkpoints, reprograms TAMs to an M2 state, may influence the efficacy of antigen presentation by DC, and affects the function of T cells, while hypoperfusion stiffens the TME that becomes a physical barrier to T cell infiltration into the tumor (123).

**Figure 5:**
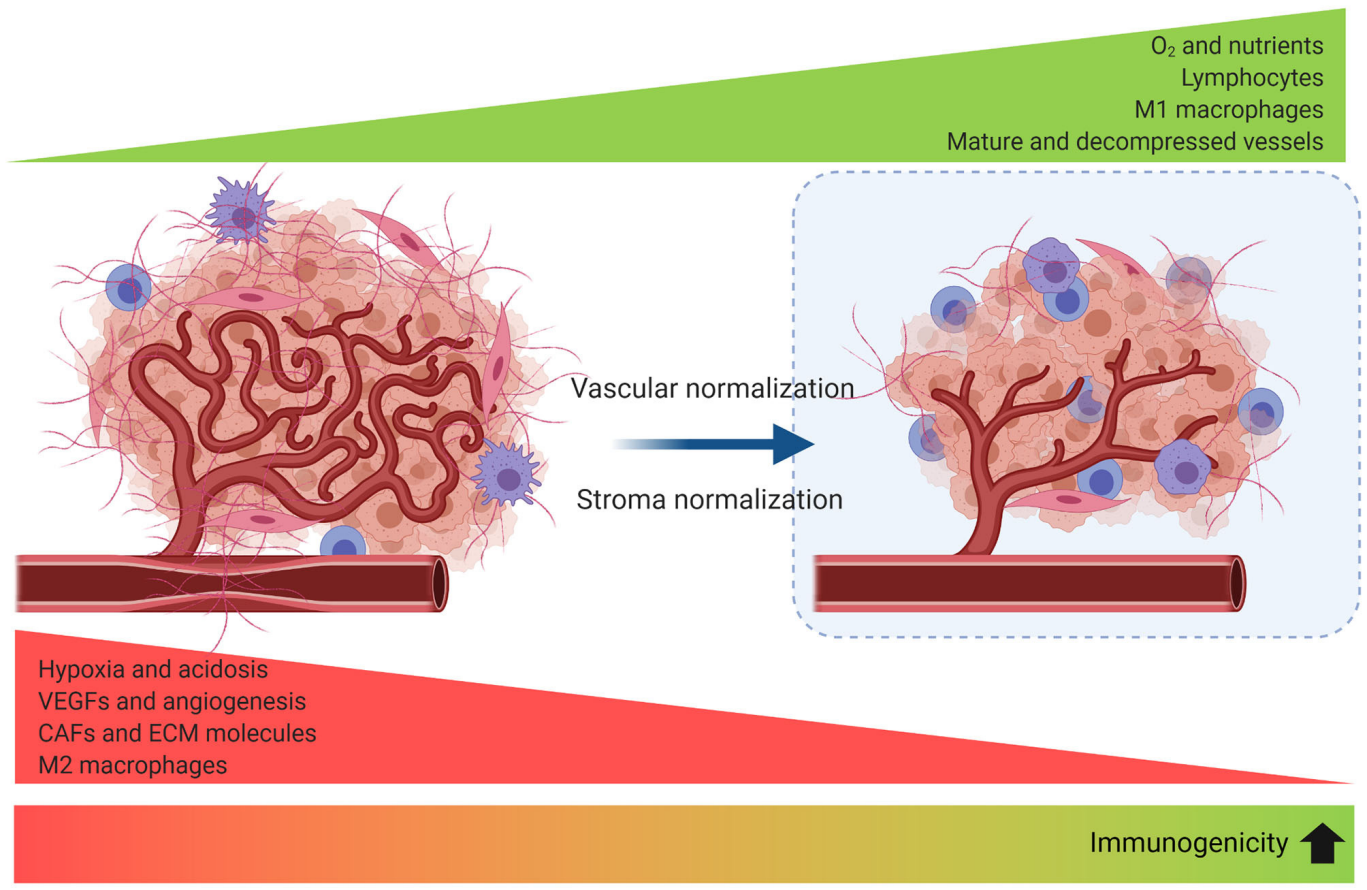
Strategies to improve tumor perfusion increase tumor immunogenicity. Angiogenesis, desmoplasia, and inflammation promote leaky and compressed tumor vessels. Vascular normalization strengthens the vessel wall reducing intercellular gaps and improving perfusion. Blood vessel decompression by depletion of CAFs or ECM reperfuses the vessel and augments perfusion. As a result, reprogramming of the TME to an immunomodulatory state enhances antitumor immunity.

An emerging field of interest investigates the synergy of immune-vascular interactions to promote an antitumor effect (124). The objective of this strategy is to induce vascular normalization that needs to be coupled to vessel decompression (to avoid vessel collapse). Restoring vessel function by normalizing tumor stroma has been evaluated in preclinical models through targeting angiotensin signaling with anti-hypertensive agents (125) or inhibiting SDF-1/CXCR4 (86) which can target CAFs and collagen/hyaluronan to decompress tumor vessels and improve perfusion and effect of the ICB. Vascular normalization can be achieved with antiangiogenic agents to improve tumor perfusion and treatment delivery, but it is dose- and time-dependent, making outcome predictions for combinations of antiangiogenics, stroma normalization and immune therapies difficult to optimize (126) (Figure 5).

Successful clinical evidence that the combination of ICB with antiangiogenic drugs has been recently reported in lung (127), renal (128, 129), and endometrial (130) cancer. However, the potential to improve the treatment outcome of this approach is under evaluation in an ongoing clinical trial, which tests the role of adding losartan (an antihypertensive angiotensin inhibitor) to chemo-radiation (delivered via SBRT) and nivolumab in pancreatic cancer patients (NCT03563248).

Apparently, any method that improves tumor perfusion is likely to enhance immunotherapy. It has been proposed that strategies that normalize the stroma would be more beneficial in tumors with abundant compressed vessels, while vascular normalization should improve perfusion in tumors with leaky vessels (131), and the combination when both co-exist. However, addressing the cause of hypoperfusion and identifying the normalization window for each tumor is challenging (126).

Since tumor perfusion is key for the efficacy of immunotherapy, perfusion markers could be used as markers for immunotherapy prediction (132).

## Future Perspectives

While recent studies have improved our understanding of mechanisms supporting immune resistance, we still have an incomplete view of how the TME works as a whole. We propose that advancements in cancer metabolism and nanotechnology represent promising areas of research that have the potential to significantly improve our understanding of immune escape in nutrient- and oxygen-poor environments which may lead to opportunities for therapeutic intervention.

A comprehensive understanding of the metabolic needs of cancer cells has been achieved during this past decade. Significantly, metabolic signatures and hypoxia within the TME impact the immune function. The fact that these findings have been translated into actionable anticancer targets provides the basis for a metabolic characterization of the TME to identify novel targets and signatures in the future. Indeed, better technologies to investigate cancer metabolism at the single-cell level without disrupting the tissue will be required to achieve a deeper understanding of the role of metabolism in cancer.

Advancements in nanotechnology have been effectively developed in cancer therapy. Innovative nanomedicines can use the conditions and characteristics of the TME to deliver therapeutics with increased precision, while providing for signal outputs that allow to follow their effects in real time. Likewise, recent advances in nanotechnology have broadened opportunities for the development of radiosensitizers in synergy with other treatment modalities. We highlight recent progress of nanotechnology between radiotherapy and immunotherapy.

### Metabolic Rewiring of the TME

The complex interplay between cellular crosstalk, interactions in the ECM and the biochemical environment within a tumor has an impact on the metabolic phenotype and polarization of immune cells. Thus, the concerted actions of different immune subsets suppress or promote growth. Solid tumors have a dynamic oxygen supply with hypoxic regions where interactions among immune cells are not well understood. Untangling these interactions might offer new potential for response prediction. Tumor infiltrating lymphocytes are at a metabolic disadvantage within the TME since tumor cells impede their access to nutrients needed for activation and acidify the TME through lactate accumulation, favoring a T_reg_ phenotype (133).

Targeting specific metabolic alterations shared by tumor cells and tumor promoting immune populations in the TME is a new strategy under evaluation. Preclinical research has focused on fatty acid metabolism as a source of metabolic plasticity in cancer cells (134), carbon metabolism to stimulate antitumor activity of macrophages (135), or targeting metabolism of ferroptosis (a form of death that relies on ROS) in tumors (136), among others. Strategies that reduce immunomodulatory metabolites are also under evaluation, which include altering the acidic microenvironment, blocking the thryptophan metabolism pathway, inhibition of adenosine within the TME (33), or avoiding lactate accumulation in the TME (137).

A coordinated approach, which takes into account tumor types and tumor biology with detailed molecular links between cancer genotypes and metabolic dependency in a longitudinal fashion, will be best suited to detect the patient populations that are most likely to benefit from metabolism-targeted therapies.

### Nanoparticle-Mediated Immunogenic Cell Death

Nanoparticles (NP) have been increasingly studied for radiosensitization. The combination of hafnium oxide NP (NBTXR3, a high-Z nanomaterial with high-level electron density that increases energy dose deposit within cells) plus radiation vs. radiation alone has recently demonstrated meaningful clinical benefit in locally advanced soft tissue sarcoma by doubling pathologic response rates (16 vs. 8%) (138). Significantly, recent research has reported that radiation-activated hafnium oxide NP can augment tumor infiltrates of CD8^+^ T cells and generate an antitumor immune response, with systemic effect on untreated tumors on the same animals in a murine model of colon cancer (139).

Newly designed hafnium-based nanoscale metal-organic frameworks (nMOFs) have demonstrated effective radioenhancement for low-dose radiation in preclinical models. The combination of nMOF-mediated radiotherapy and PD-L1 blockade extended the local therapeutic effects of radiation to distant tumors via systemic antitumor immunity. This powerful platform can minimize toxic effects by lowering the administered dose of radiation; it can be redesigned for rational tuning and can significantly strengthen the effect of immunotherapy for treatment of non-immunogenic tumors (140, 141).

## Conclusion

Evolution in the technological delivery of radiation and precision surgery parallels the rapid progress in immune biology that identifies novel strategies to enhance the antitumor immune response. In this setting, alterations in the TME could become especially relevant to optimize treatment immunogenicity and enhance patient outcome.

Defining the individual response of tumors to surgery and radiation offers the possibility to design innovative treatment strategies and re-adapt treatment to new emerging targets. This could have a major impact since it potentially represents a novel way to enhance local and systemic treatments.

## Author Contributions

The structure of the manuscript was compiled by all authors. SB and ML wrote the first draft. AS-G and SB assembled the figures. All authors reviewed and approved the final version of the manuscript.

## Conflict of Interest

The authors declare that the research was conducted in the absence of any commercial or financial relationships that could be construed as a potential conflict of interest.

## Abbreviations

4-1BB: tumor necrosis factor receptor superfamily member 9, CD137
A2AR: adenosine receptor A2
APC: antigen-presenting cells
*ATM*: ataxia telangiectasia mutated
ATP: adenosine triphosphate
CAFs: cancer-associated fibroblasts
CCL2: CC chemokine receptor 2
CD28: cluster of differentiation 28
CD39: cluster of differentiation 39
CD73: cluster of differentiation 73, ecto-5′-nucleotidase
CD80: cluster of differentiation 80
CD86: cluster of differentiation 86
cGAS: cyclic GMP-AMP synthase
CHK1: checkpoint kinase 1
CSF-1: colony stimulating factor 1
CSF-1R: colony stimulating factor 1 receptor
CTLA-4: CTL antigen 4
CXCL1: C-X-C motif chemokine ligand 1
CXCL2: C-X-C motif chemokine ligand 2
CXCL10: C-X-C motif chemokine ligand 10
CXCL16: C-X-C motif chemokine ligand 16
CXCR4: C-X-C chemokine receptor type 4
DAMPs: damage-associated molecular patterns
DC: dendritic cells
DDR: DNA damage response
DNA-PK: DNA-dependent protein kinase
ECM: extracellular matrix
HIF-1α: hypoxia-inducible factor-1α
HLA: human leukocyte antigen
HMGB1: high mobility group box 1
HSP90: heat shock protein 90
ICB: immune checkpoint blockade
IFN: interferon
IFN-γ: interferon gamma
IL-4: interleukin 4
IL-6: interleukin 6
IL-10: interleukin 10
IL-13: interleukin 13
IL-35: interleukin 35
irAEs: immune-related adverse events
*KPNA2*: karyopherin subunit alpha 2
LAG3: lymphocyte-activation gene-3
LUM: LUM imaging system (Lumicell Inc.)
LUM015: cathepsin activatable fluorescent probe
MDSCs: myeloid-derived suppressor cells
MHC-I: major histocompatibility complex-I
NF-κβ: nuclear factor-kappa beta
NK: natural killer
nMOFs: nanoscale metal-organic frameworks
NP: nanoparticles
NSCLC: non-small cell lung cancer
OX-40: tumor necrosis factor receptor superfamily member 4, CD134
PARP: poly(ADP-ribose) polymerase
PD-1: programmed death-1
PD-L1: programmed death-ligand 1
ROS: reactive oxygen species
SBRT: stereotactic body radiotherapy
SDF-1: stromal cell-derived factor-1
SRS: stereotactic radiosurgery
STING: stimulator of interferon genes
TAMs: tumor-associated macrophages
TGFβ: transforming growth factor β
Th1: T helper type 1
Th2: T helper type 2
TIGIT: T cell immunoglobulin and ITIM domain
TIM-3: T cell immunoglobulin and mucin domain-3
TLR: toll-like receptors
TLR3: toll-like receptor 3
TLR7/8: toll-like receptors 7/8
TLR9: toll-like receptor 9
TMB: tumor mutational burden
TME: tumor microenvironment
TNFα: tumor necrosis factor α
Treg: regulatory T cells
Trex1: three-prime repair exonuclease 1
VEGF: vascular endothelial growth factor.
